# Comprehensive analysis of m6A regulators associated with immune infiltration in Hepatitis B virus-related hepatocellular carcinoma

**DOI:** 10.1186/s12876-023-02873-6

**Published:** 2023-07-28

**Authors:** Zhen Zhang, Wenhui Gao, Zhuo Liu, Shuxian Yu, Huiying Jian, Zongwei Hou, Puhua Zeng

**Affiliations:** 1 Department of Oncology, Affiliated Hospital of Hunan Academy of Traditional Chinese Medicine, Changsha, 410006 P.R. China; 2 School of Chinese Medicine, Hunan University of Chinese Medicine, Changsha, 410208 P.R. China

**Keywords:** HBV-related HCC, m6A, Immune infiltration, Cancer treatment, Risk model

## Abstract

**Background:**

N6A methylation (m6A) is a significant epigenetic modification that critically impacts post-transcriptional regulation and tumor occurrence and development. While previous studies have identified a role for epigenetic regulation in hepatocellular carcinoma (HCC), the potential function of the m6A cluster in Hepatitis B virus (HBV)-related HCC remains unclear.

**Methods:**

The related information was downloaded from The Cancer Genome Atlas (TCGA) and Gene Expression Omnibus (GEO). Based on the expression of 20 m6A regulators, we comprehensively evaluated the m6A clusters and systematically explored the correlation between these clusters and immune cell infiltration characteristics of the tumor microenvironment (TME). The patients were divided into low- and high-m6A score groups. Then, the immune cell infiltration, chemokines, and cytokines levels, and drug sensitivity were further explored between the two groups.

**Results:**

The m6A cluster predicted a better prognosis that was accompanied by increased immune cell infiltration. Using these results, an m6A score was established that could predict overall survival, immune checkpoints, and clinical treatments for patients with HBV-related HCC. This study demonstrated that m6A modifications affected tumorigenesis, TME, and the prognosis of patients with HBV-related HCC.

**Conclusion:**

A comprehensive assessment of m6A patterns could improve the current understanding of immune cell infiltration patterns and inform the development of individualized cancer treatments.

**Supplementary Information:**

The online version contains supplementary material available at 10.1186/s12876-023-02873-6.

## Introduction

Hepatocellular carcinoma (HCC) is one of the most malignant cancers, with over 700,000 related deaths occurring globally each year [1]. According to recent etiological studies, viral hepatitis infection, alcohol abuse, and aflatoxin exposure are the primary risk factors for HCC [2–4]. Of these, chronic hepatitis B virus (HBV) infection is the most prevalent, accounting for more than 80% of HCC cases in China and other countries [5, 6].

Conventional treatments, such as surgery, transcatheter arterial chemoembolization (TACE), and radiotherapy, make little difference to prognosis and are associated with significant side effects. Immunotherapy has been shown to improve the prognosis of malignant diseases by activating the immune system to identify and eliminate tumor cells [7]. improves disease control in over 40% of patients with advanced HCC [8]. However, for several reasons, only a minority of patients can benefit from immunotherapy. One of the main reasons is that cytokines and immunosuppressive cells in the tumor microenvironment (TME) can promote immune escape [9]. Thus, it is crucial to define the regulatory mechanisms and novel markers of HBV-related HCC in order to guide and predict appropriate immunotherapeutic responses.

RNA modification is a form of post-transcriptional modification of gene expression. More than 170 types of RNA modification have been identified [10]. N6-methyladenosine (m6A), one of the most prominent and abundant methods, includes the methylation of N6 RNA adenosine. To date, 20 m6A regulators have been identified, comprising three kinds: methyltransferases or “writers” (METTL3, WTAP, RBM15, KIAA1429, METTL14, and ZC3H13), binding proteins or “readers” (THDF1/2/3, YTHDC1/2, eIF3, IGF2BP1/2/3, HNRNPA2B1, FMR1, and LRPPRC), and demethylases or “erasers” (FTO and ALKBH5). Recent studies indicate that m6A modification is associated with several bioprocesses, including immunity, metabolism, and proliferation [11, 12]. It is well known that m6A modification is a dynamic process that modulates RNA translation, degradation, and nuclear export. Recent studies have associated the dysfunction of m6A regulators with serious cancer processes, including cell death, drug resistance, and immunomodulatory abnormalities [13, 14].

METTL3 can promote HBV replication and apoptosis in liver cells, as well as enhance the production of pro-inflammatory cytokines [15]. METTL3 and METTL14 are recruited by HBV X protein (HBx). Knockout of FTO and ALKBH5 can downregulate the expression of HBV-related proteins [16], and YTHDF2 can bind to ISG20 to promote HBV RNA decay and HBV transcript degradation. When HBV-infected cells are silenced, HBV RNA becomes less sensitive to ISG20-mediated degradation [17]. HBx regulates viral transcription and replication, and its interaction with METTL3/14 promotes HBV cDNA and tensin homolog chromosomal loci recruitment during HBV infection [18]. HBx protein also upregulates expression of the Writer protein, METTL3, and increases m6A modification of circ-ArL3. The Reader protein, YTHDC1, then binds to the m6A modified circ-ArL3 for reverse splicing and biogenesis [19].

Research has indicated that dysfunction of m6A regulators has a significant impact on the progression of human cancers [20, 21]. As reported, METTL3 is highly expressed in cancerous tissue [22]. METTL3 can also promote SOCS2 m6A methylation, causing degradation of SOCS2 and leading to tumorigenesis [22]. Another m6A methyltransferase, METTL14, is associated with aberrant m6A modification in HCC tissue, and low METTL14 expression promotes HCC metastasis both in vivo and in vitro [23].

While anti-tumor effects are characterized by the highly coordinated interaction of multiple tumor suppressors, recent studies have typically assessed only one or two factors simultaneously. Thus, a comprehensive assessment of how multiple m6A regulators mediate immune cell infiltration would provide greater insight into the immune regulation of tumors. The current study systematically analyzes m6A regulator expression patterns within the tumor immune landscape. Two m6A modification clusters were identified, and an m6A scoring system was established to quantify patients with HBV-related HCC in order to predict and guide specific immunotherapy.

## Materials and methods

### Data collection

Transcriptome profiling data was obtained from The Cancer Genome Atlas (TCGA) (https://www.cancer.gov/about-nci/organization/ccg/research/structural-genomics/tcga, accessed on 12 November 2021) and Gene Expression Omnibus (GEO, GSE14520) (https://www.ncbi.nlm.nih.gov/geo/, accessed on 12 November 2021). The GSE14520 dataset was based on the GPL571 and GPL3921 platforms. The TCGA database includes 103 tumor tissues and 50 adjacent nontumor tissues while the GEO database consists of 212 tumor tissues and 241 non-tumor tissues. TCGA data were normalized using the fragments per kilobase of exon per million reads mapped (FPKM) method, followed by a log_2_ transformation. A batch correction was performed on the two databases. Since these data are publicly accessible, no approval from an ethics committee was requested for access. R (version 4.1.1) and R Bioconductor packages were used to determine the ratio of somatic mutations and conduct the CNV analyses.

### Clustering of m6A regulators by consensus cluster

Unsupervised agglomerative cluster analysis was performed using the “ConsensusClusterPlus” package in R to divide HBV-related HCC patients into two different m6A subgroups with distinct m6A subtypes [24]. The “Principal Component Analysis” package was employed to analyze subtype-specific gene expression.

### Gene Set Variation Analysis

We employed the “Gene Set Variation Analysis” (GSVA) package in R to explore the biological processes of each m6A subtype [25]. The well-defined biological pathways and functions were drived from the Hallmarker gene set “c2.cp.kegg.v7.2.symbols.gmt” (downloaded from MSigDS database v7.4) [26].

### Exploration of immune cell infiltration and immune-related function

Twenty-three immune cell types’ abundance and activity levels were obtained from published signature gene lists and quantified by “Single Sample Gene Set Enrichment Analysis” (ssGSEA) in the GSVA package [27]. In this study, nine types of innate immune cells, including macrophages, mast cells, neutrophils, nature killer (NK) cells, CD56dim NK cells, CD56bright NK cells, dendritic cells (DCs), plasmacytoid dendritic cells (pDC), immature DCs (iDC), and eight types of adaptive immune cells, including B cells, T cells, CD8 + T cells, T follicular helper (TFH), T helper (Th)1, Th2, Th17, and Treg cells, comprised these signatures. Additionally, ssGSEA was employed to investigated the relationship between distinct m6A subtypes and immune-related pathways in the HBV-related HCC expression profile. A Gaussian fitting model was employed to estimate the bio-similarity of the infiltrating immune cells and immune related functions.

### Differently expressed genes (DEGs) in the m6A regulator subtypes

An empirical Bayesian approach was conducted using the limma R package to identify the DEGs in the two m6A clusters. Based on consensus clustering, the patients were divided into three groups to evaluate m6A regulator gene expression. The DEGs screening cutoff for significance was set at *P* < 0.05. The random forest method was used to remove redundant genes DEGs using the “Hmisc” package. The functional annotation of these DEGs was carried out using clusterProfiler, which incorporated both Gene Ontology (GO) and Kyoto Encyclopedia of Genes and Genomes (KEGG) analysis.

### Assessment of the m6A signature

A rating system was constructed to define m6A modification patterns that were associated with malignancy during HBV-related HCC. The m6A signature, or m6A score, was established by extracting DEGs from the m6A cluster, normalizing them in all HBV-related HCC samples, and identifying any overlapping genes. The consensus clustering algorithm was used to obtain a gene cluster and an in-depth analysis of any genes associated with a significant prognosis was performed. Individual m6A scores were calculated as described previously, using the following Eqs.  [28, 29]:

$${\text{m6Ascore}} = \sum {\left( {PC{1_i}} \right)} + \sum {\left( {PC{2_i}} \right)}$$

where i represents the expression of m6A phenotype-relater genes.

### Patients and tissue specimens

Ten HBV-related HCC tissue and precancerous tissue samples were obtained from the Affiliated Hospital of Hunan Academy of Traditional Chinese Medicine between March 2019 and March 2020. Patients were not directly involved in the study. The samples were used in accordance with the regulations from the ethics committee of the Affiliated Hospital of the Hunan Academy of Traditional Chinese Medicine. Included patients were between 18 and 70 years of age, had adequate function of their major organs (including the heart, liver, and kidneys), and had not received medical treatment before surgery. All patients were carefully screened and only included if they did not have serious medical or surgical diseases, including diabetes, hypertension, and other cancers. Patients were excluded if they had received chemotherapy, radiotherapy, or transarterial chemoembolization therapy.

### qRT-PCR

Total tissue RNA was extracted using TRIzol reagent (Tiangene, China) according to the manufacturer’s protocol and transcribed into cDNA. Using qRT-PCR was carried out and relative mRNA expression was determined using the 2^−ΔΔCt^ method. The primers were as follows in Table 1.

**Table 1:** Primers sequence of m6A regulators

GAPDH-F	ATCCCATCACCATCTTCC	RBMX-F	TCCTTCAGCCTCGTTCC
GAPDH-R	ATGACCCTTTTGGCTCCC	RBMX-R	GGGGTGACAATGGGTTC
METTL3-F	TTGCCCACTGATGCTGT	ZCCHC4-F	CGGAGGTTTTATGCCTGT
METTL3-R	GGAGACCTCGCTTTACCTC	ZCCHC4-R	TTCGGTTATGAGCTTCTCG
IGFBP1-F	GCACGGAGATAACTGAGGA	YTHDF1-F	GCACACAACCTCCATCTTC
IGFBP1-R	ACATGGAGAGCCTTCGAG	YTHDF1-R	ACTGGTTCGCCCTCATT
IGFBP2-F	GGGGAGTGCTGGTGTGTG	YTHDF2-F	GCAAGCAATGTTCCAAAAG
IGFBP2-R	GCTGGCTGCGGTCTACTG	YTHDF2-R	GCAATATCAGCCCAAGATG
IGFBP3-F	AGCGGGAGACAGAATATGG	YTHDF3-F	CGCATCTGCCCTTATACTCT
IGFBP3-R	TTTGGAAGGGCGACACT	YTHDF3-R	TCATTCAGTCTTCTGTGCCTT
FTO-F	TGAGGTCGAGTTTGAGTGG	FMR1-F	CAAAAGTCCAGAGGGTGTTAG
FTO-R	TGCTTCCAGTTGAGCCAT	FMR1-R	AAGAACAGTGGCATTAGCG
WTAP-F	TTCCATTCAACAGGCACA	HNRNPA2B1-F	AAAGTTGTAGGTTGGCTGTTG
WTAP-R	CCCCATTAACACCAACGA	HNRNPA2B1-R	GCCCTGAGTATCACATTCCT
RBM15-F	CTCGGGATAGGACACCAC	LRPPRC-F	GCAAGTTAGGCGGGATT
RBM15-R	TCGTAAGCAGGCACAGAA	LRPPRC-R	AATGCCTGGATGACACG
RBM15B-F	AGCCAAAACACGAGACCAA	ZC3H13-F	TCACACAGAAAATGCCACA
RBM15B-R	CATAGACGTGGGGAAGCAG	ZC3H13-R	CGGTTACTCCGATTGCTC

‘F’, indicates forward; ‘R’, indicates reverse

### Statistical analysis

One-way ANOVA and Kruskal-Wallis tests were used to compare the subgroups [30]. The “survminer” R package was employed to determine the cut points for each data set by distinct groups and the “surv-cutpoint” package was used to dichotomize the m6A score. Based on maximum grade, high and low m6A score groups were created to decrease the calculated batch effect. The “maftools” R package was used to plot the variation of m6A regulators in chromosome pairs. All statistical *p* values were bilateral, with *p* < 0.05 considered statistically significant.

## Results

### M6A regulators are likely to have a significant effect on HBV-related HCC

**Fig. 1:**
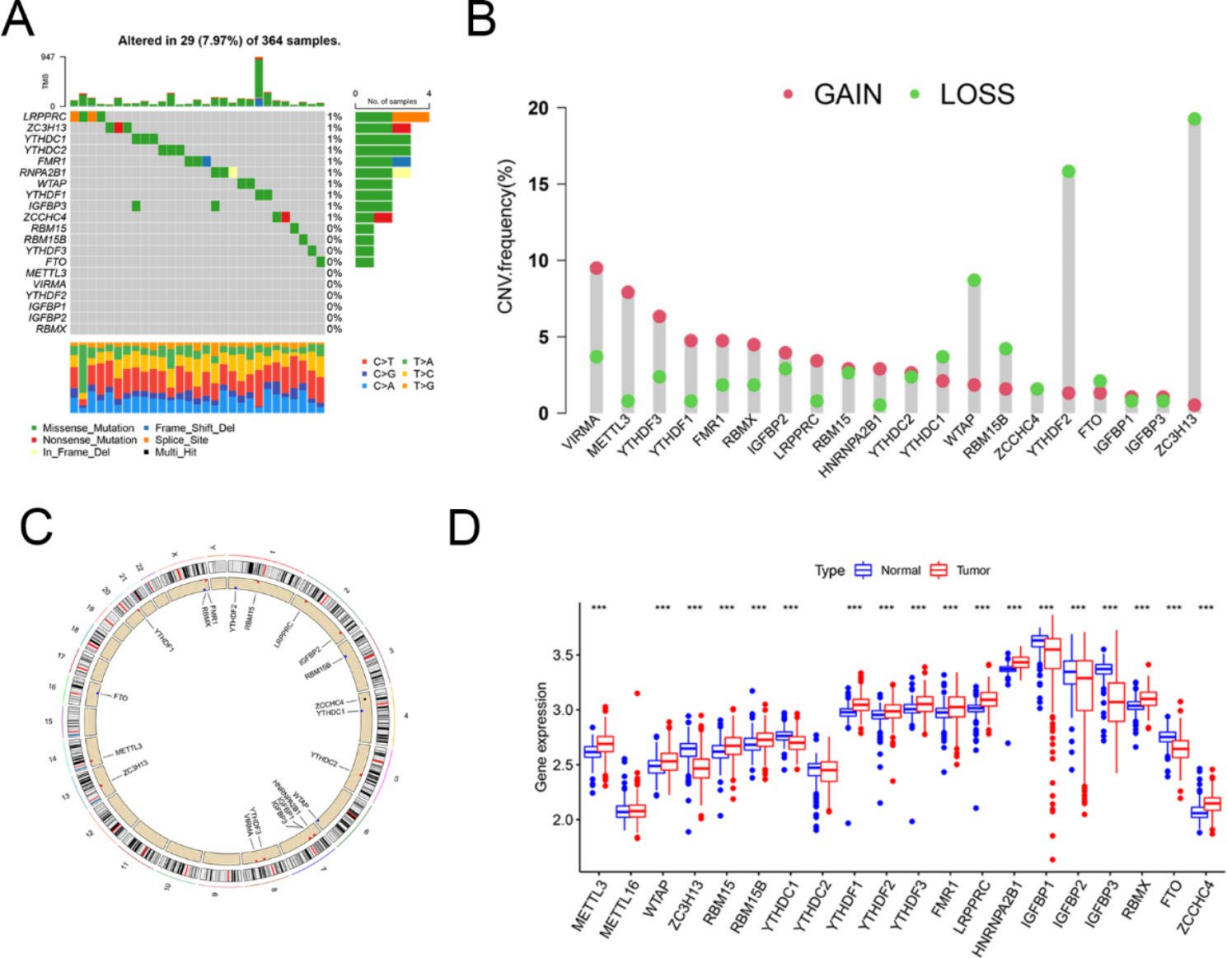
Overview of N6-methyladenosine (m6A) regulators in patients with HBV-related HCC. (**A**) The waterfall of m6A gene somatic mutations and mutation types. (**B**) The frequency of CNV of the m6A regulators. Green dots represent the deletion of CNV; pink dots represent a gain of CNV. (**C**) Locations of m6A gene mutations on the chromosomes. (**D**) Differential m6A regulator gene expression between normal and HBV-related HCC. ***P < 0.001

Twenty m6A regulators, including 12 readers, 7 writers, and 1 eraser, were identified in the TCGA and GEO cohorts. The correlation network provided interactive information among the m6A regulators. The ratio of somatic mutations and CNV for the m6A regulators was also investigated. Of the 364 samples, 29 (7.9%) had somatic mutations (Fig. 1A). All of the genes had significant CNV (Fig. 1B), with 11 having a higher copy number and 8 having a lower copy number. ZC3H13 had a range of CNV deletion frequencies. The location of CNV alterations on the m6A regulator chromosome is shown in Fig. 1C. To determine whether CNV and somatic mutations influenced m6A regulator expression in HBV-related HCC, m6A regulator expression was compared between normal and malignant tissues. Twelve of the genes had higher expression and six had lower expression in malignant samples (Fig. 1D). Importantly, m6A regulator expression showed significant heterogeneity between normal and HBV-related HCC tissues, demonstrating that they are likely to have a significant effect on HBV-related HCC.

### Two identified m6A methylation modification patterns had different overall survival rate

**Fig. 2:**
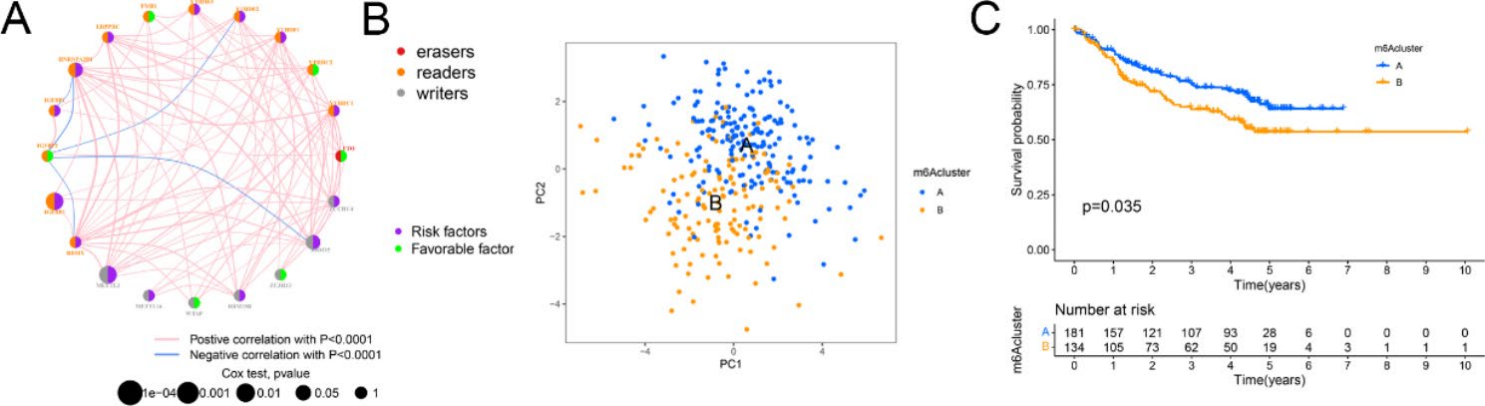
Identification of m6A regulator modification. (**A**) The communication of 20 m6A regulators in HBV-related HCC. The color represents the RNA modifications. Red, erasers; orange, readers; gray, writers. Purple circles represented risk factors. The green circle represents favorable factors. The pink line represents a positive correlation between m6A regulators with *P *< 0.0001; the blue line represents a negative correlation between m6A regulators with *P *< 0.0001. (**B**) The significant difference between the two m6A clusters was marked. (**C**) Kaplan-Meier curves of overall survival between m6A clusters A and B

Patients in the TCGA and GEO datasets with available survival and related clinical information were enrolled in the study. The regulatory network shows the interactions between m6A regulators and their prognostic significance for HBV-related HCC patients (Fig. 2A). The results indicate that the interconnection between the reader, writer, and eraser regulators may play an important role in creating distinct m6A modification patterns and influence the prognosis of HBV-related HCC patients.

According to these 20 m6A regulators, the R package ConsensusClusterPlus was employed to classify patients into qualitatively distinct m6A clusters. Patients were divided into two clusters using unsupervised clustering: cluster A (184 cases) and cluster B (114 cases) (Fig. 2B). Cluster A had a higher overall survival rate than cluster B (Fig. 2C).

### M6A clusters impact the landscape of the tumor immune microenvironment (TIME)

**Fig. 3:**
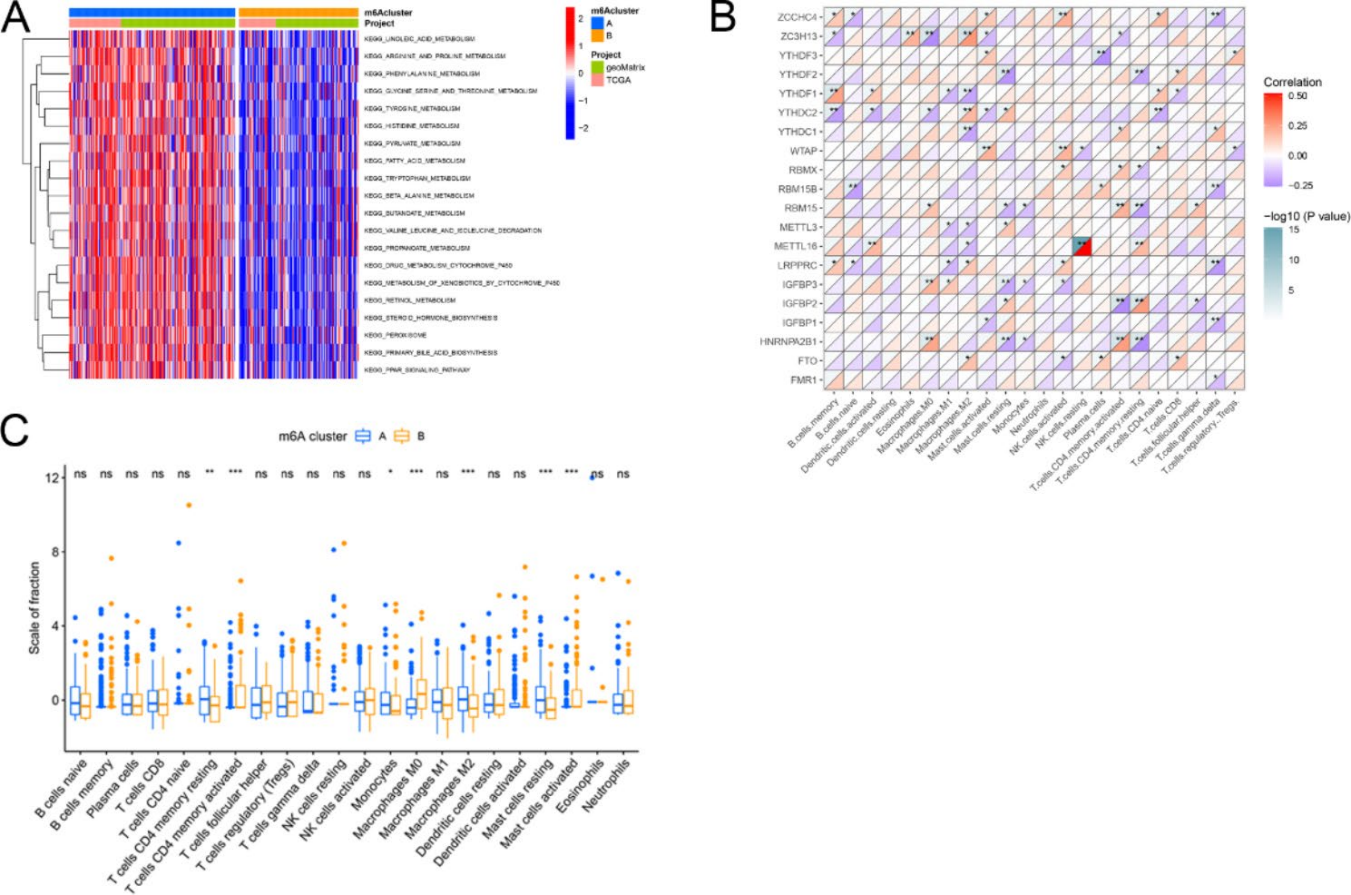
Comparison between the function and immune infiltration of the m6A regulator clusters. (**A**) Biological function analysis of different m6A clusters. (**B**) The heatmap of consensus clustering in the TCGA and GEO cohort. Clinical information includes age, gender, and survival state. (**C**) Correlation between the m6A regulator and immune cell infiltration. Purple represents a negative correlation and red represents a positive correlation

GSVA enrichment analysis of the KEGG gene sets was performed to investigate the biological functions and associated pathways of the m6A clusters. The difference between the two m6A clusters was primarily associated with metabolic pathways, including those involving linoleic acid, arginine and proline, phenylalanine, glycine serine and threonine, and tyrosine (Fig. 3A). Then, Spearman’s correlation was employed to investigate the association between m6A gene expression and TIME infiltration. The m6A regulators were found to differentially impact and coordinate in the regulation of immune cell infiltration (Fig. 3B). There was a difference in TIME infiltration between the two m6A clusters. Lymphocytes, including activated CD4 + memory resting T cells, monocytes, M2 macrophages, and resting mast cells, were primarily enriched in m6A cluster A, while CD4 + memory activated T cells, activated NK cells, and mast cells were primarily enriched in cluster B (Fig. 3C).

### Generation of m6A regulator signatures and biological function

**Fig. 4:**
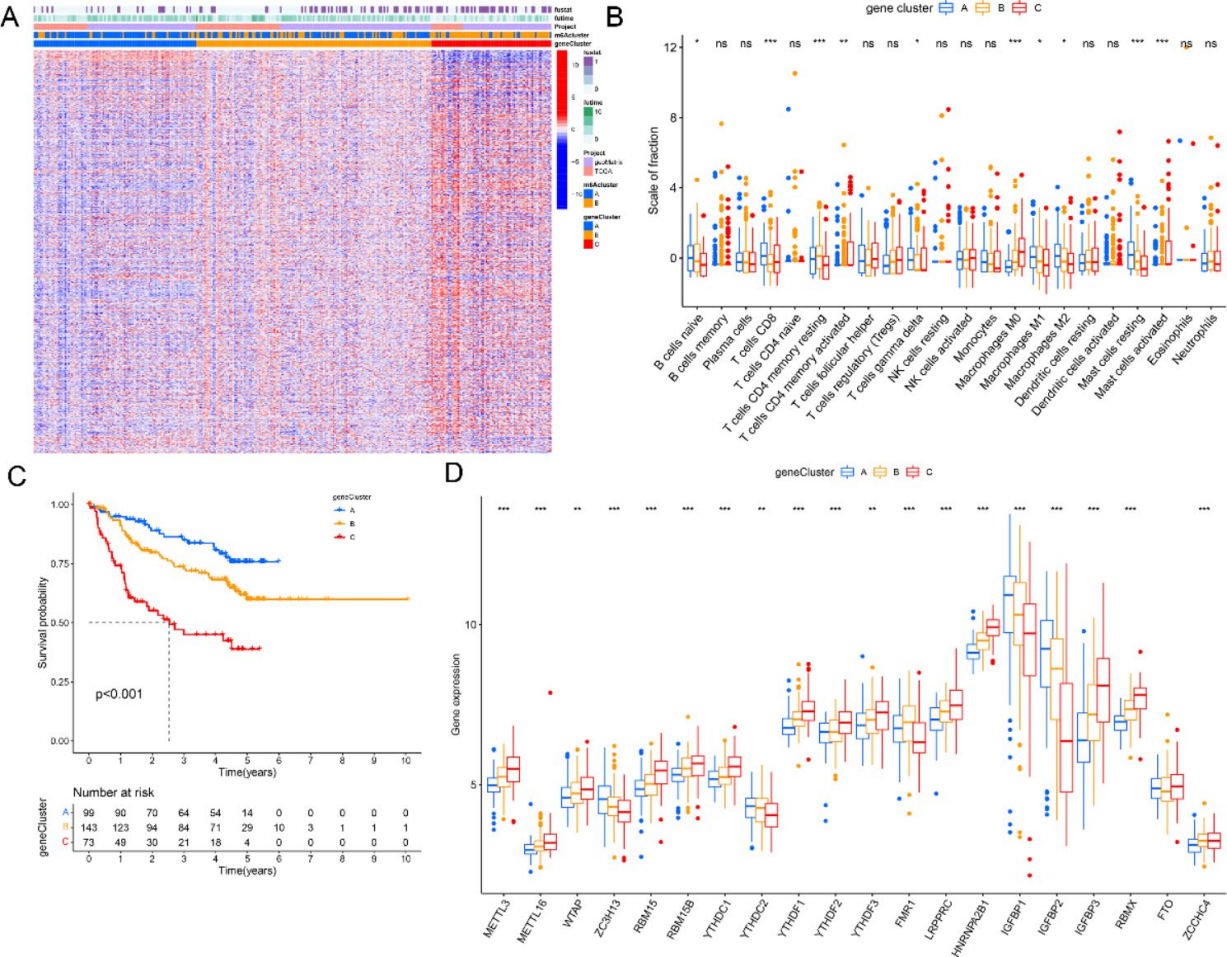
Identification of gene cluster and function analysis. (**A**) Heatmap of gene expression in the two m6A clusters. (**B**) Infiltration level of immune cells in three gene clusters. (**C**) Survival analysis in the three clusters. (**D**) M6A regulator gene expression in the three clusters

**Fig. 5:**
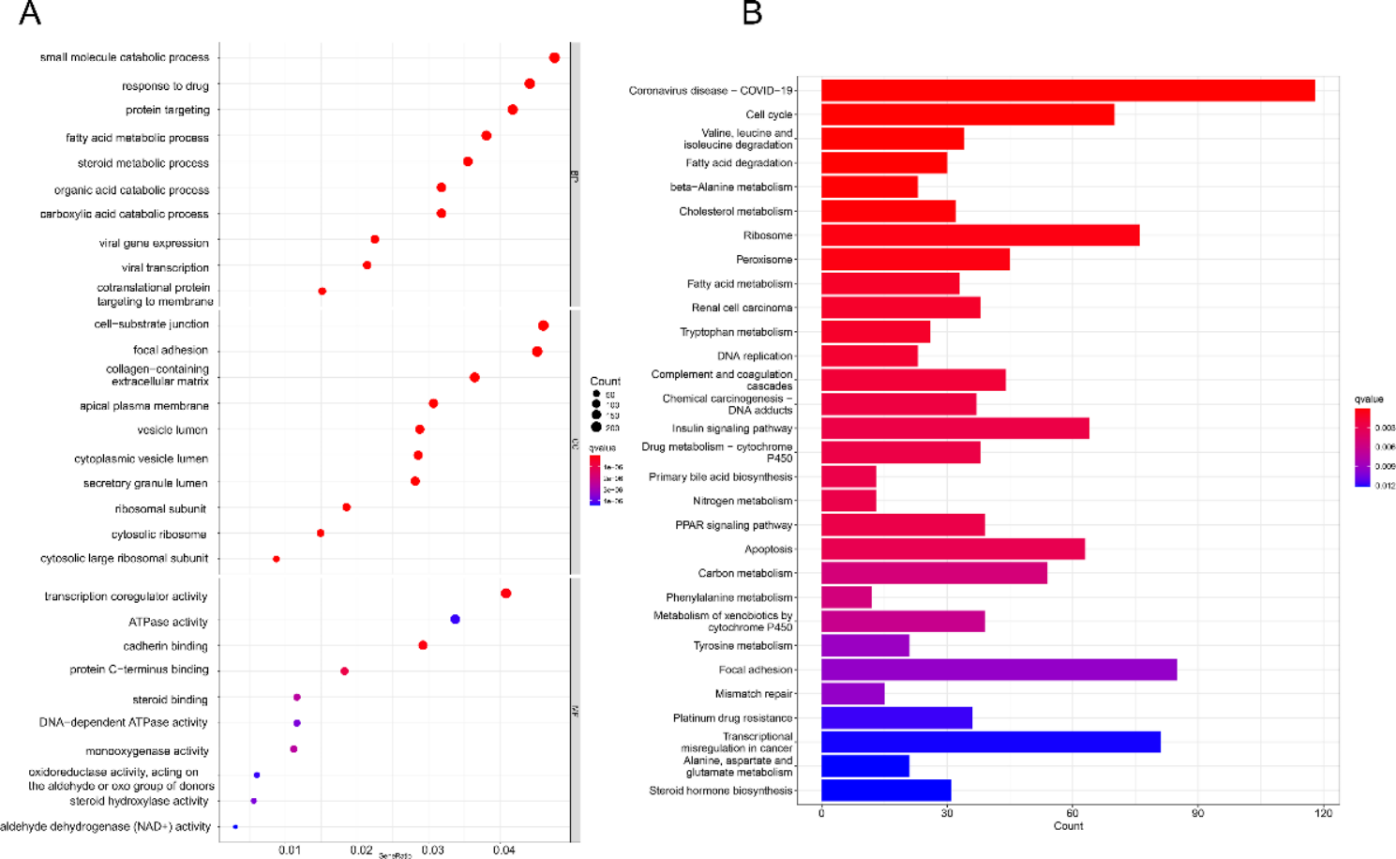
Function analysis of gene cluster. (**A**) GO enrichment analysis of DEGs. (**B**) KEGG [26] enrichment analysis of DEGs

To further investigate the biological function and in-depth mechanism of the m6A regulators, transcriptional expression changes were assessed in the two m6A clusters in HBV-related HCC patients. In total, 4,848 DEGs were identified in the two m6A clusters, and 347 DGEs associated with disease prognosis were selected for further study (Fig. 4A). The unsupervised clustering method was used to divide patients into three gene clusters, and immune cell infiltration was measured in each cluster. Naive B cells, CD8 + T cells, γδ T cells, M1 macrophages, M2 macrophages, and resting mast cells were significantly enriched in the m6A gene cluster A, indicating that the advanced prognosis outcome may be a result of higher immune cell infiltration (Fig. 4B). K-M analysis indicated that patients in the m6A gene cluster C had poor prognostic outcomes, while those in the m6A gene cluster A had positive outcomes (Fig. 4C). The expression of the 20 regulators in different m6A gene clusters was explored, and results showed that 19 of 20 m6A regulators were differentially expressed in each gene cluster (Fig. 4D). GO and KEGG enrichment analysis indicated that these DEGs were primarily involved in the cell cycle, valine, leucine, and isoleucine degradation, and fatty acid degradation (Fig. 5A and B).

### Established m6A score has good predictive power

**Fig. 6:**
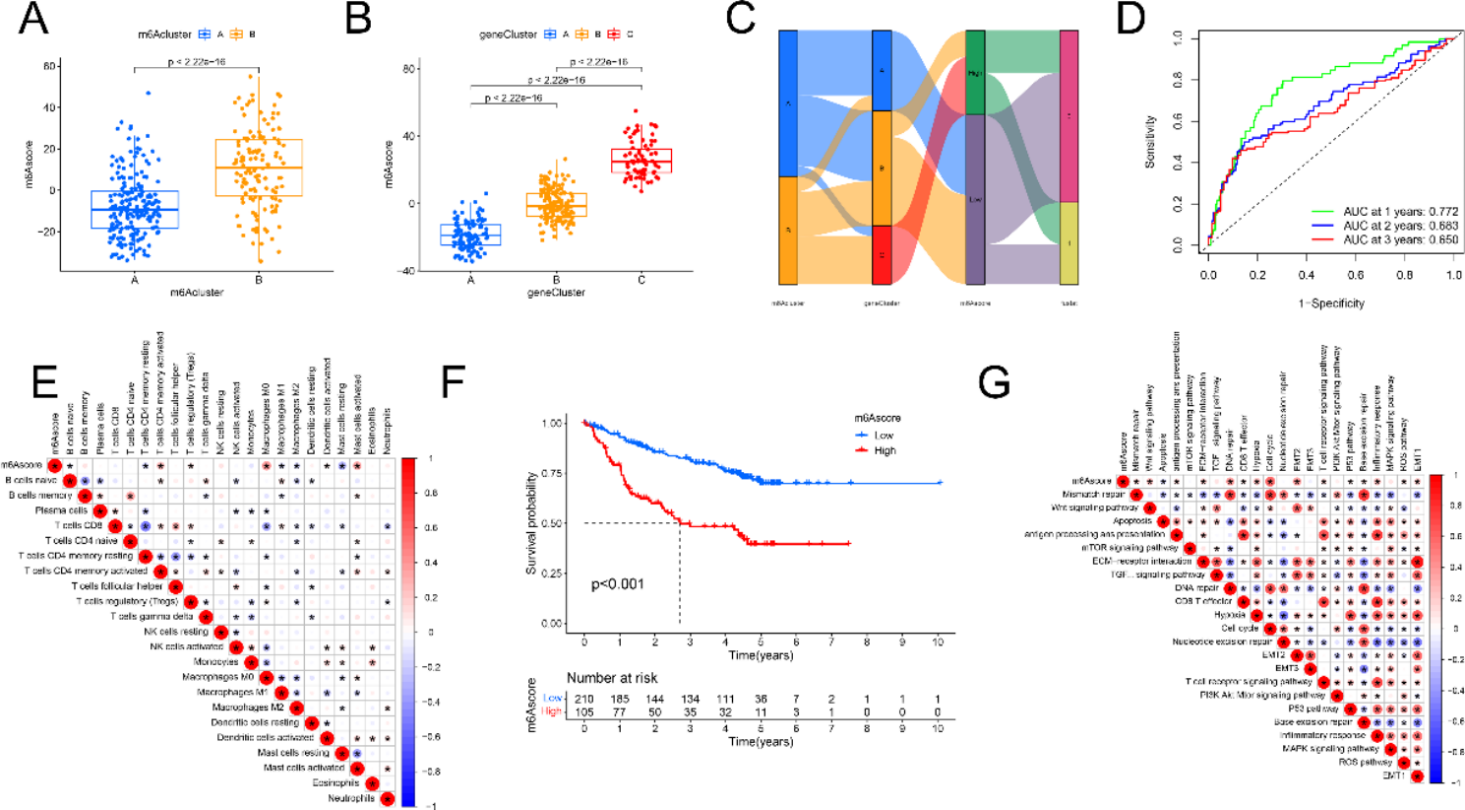
Identification of m6A score and analysis of its genetic features. (**A**) Correlation between m6A clusters and m6A score. (**B**) Correlation between gene clusters and m6A scores. (**C**) Alluvial diagram of the relationship of m6A clusters, gene clusters, and m6A scores. (**D**) AUC of 1-, 3-, 5- years. (**E**) The relationship between m6A score and the immune cell infiltration. (F) OS analysis of patients in the m6A score subgroups. (**G**) The relationship between m6A score and key biological processes

Findings from this study indicate that m6A regulators play an important role in prediction of prognosis and modulating TIME. in patients with HBV-related HCC. However, these results are only reflective of overall patients with HBV-related HCC, rather than the heterogeneity and complexity of individual m6A regulators. Using the confirmed m6A regulators, a scoring scheme was developed to quantify the m6A score of individual patients. Each patients had a distinct m6A score (Fig. 6A). M6A cluster B had a higher m6A score, suggesting that lower scores may be associated with immune activation (Fig. 6B). In addition, m6A gene cluster A had a significantly lower m6A score, and cluster C had an increased m6A score. These results are consistent with previous findings that a lower m6A score in HBV-related HCC correlates with poor prognosis outcomes. The alluvial diagram confirmed that m6A cluster A was associated with a lower m6A score, while m6A cluster B exhibited a higher m6A score (Fig. 6C). Spearman’s correlation analysis was used to explore the immune patterns of m6A regulators. AUC of 1-, 3-, 5- years of m6A score was shown in Fig. 6D. M6A score was negatively associated with naïve B cells, CD4 + memory resting T cells, resting mast cells, M1 macrophages, and M2 macrophages, while positively related to T cells CD4 memory active, macrophage M0, and activated mast cells (Fig. 6E). This analysis also showed that the m6A score was closely related to tumor stage and survival status.

Survival analysis of m6A regulators indicated that 13 of 20 genes could influence the OS of HBV-related HCC patients (Supplementary Fig. 1). Higher expression of FMR1, YTHDC2, and ZC3H13 was associated with a better prognostic outcome. In contrast, low expression of HNRNPAIB1, IGFBP1, IGFBP3, METTL3, METTL16, RBM15, RBMX, YTHDC1, YTHDC2, YTHDF1, and YTHDF2 correlated with an improved prognosis (Fig. 6F). Spearman’s correlation analysis was also used to assess the functional patterns of m6A regulators. M6A score was positively associated with mismatch repair, wnt signaling pathway, antigen processing and presentation, and EMT signaling (Fig. 6G).

### Correlation between m6A score and clinical characteristics, chemokines, and cytokines prediction, and drug sensitivity analysis of m6A scores

The results of this study suggest that m6A regulators play a crucial role in modulating the prognosis and tumor microenvironment of patients with HBV-related HCC. However, the study only reflects the overall picture of patients with HBV-related HCC and does not consider the heterogeneity and complexity of individual m6A regulators. To address this, a scoring scheme was developed to quantify the m6A score of individual patients, using the confirmed m6A regulators. Each m6A cluster was associated with a distinct m6A score, and cluster B had a higher score, suggesting that lower scores may be associated with immune activation. M6A gene cluster A had a significantly lower m6A score, and cluster C had an increased m6A score, consistent with previous findings that a lower m6A score in HBV-related HCC correlates with poor prognosis outcomes. The alluvial diagram confirmed that m6A cluster A was associated with a lower m6A score, while m6A cluster B exhibited a higher m6A score. Spearman’s correlation analysis was used to explore the immune patterns of m6A regulators, showing that the m6A score was negatively associated with naïve B cells, CD4 + memory resting T cells, resting mast cells, M1 macrophages, and M2 macrophages, while positively related to T cells CD4 memory active, macrophage M0, and activated mast cells. This analysis also showed that the m6A score was closely related to tumor stage and survival status.

**Fig. 7:**
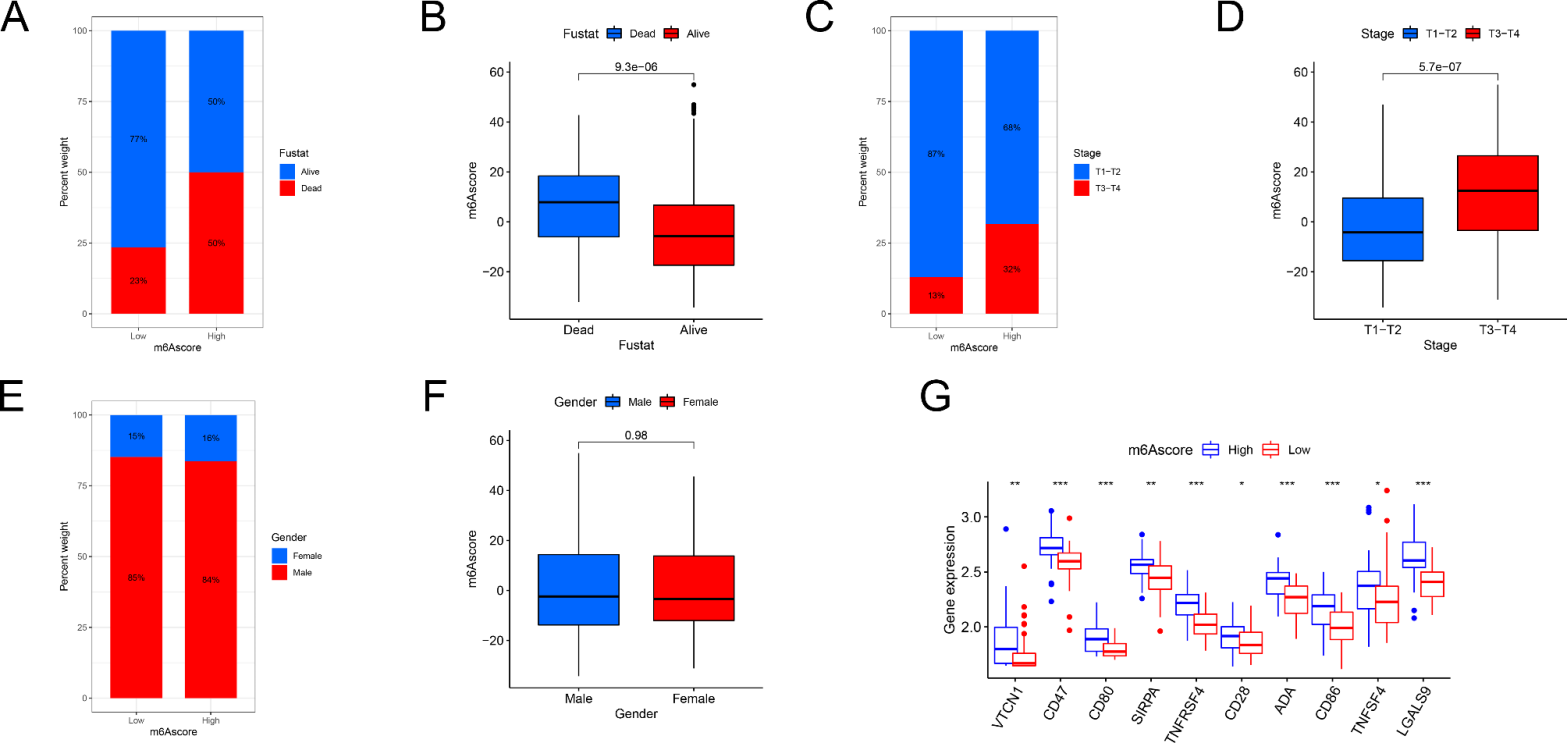
Correlation between m6A score and clinical characteristics. (**A**) The proportion of patients alive and dead in different m6A groups. (**B**) M6A scores of alive and dead patients. (**C**) The proportion of patient stage T1-2 and T3-4 under different m6A score groups. D. The difference in m6A score between patient stages T1-2 and T3-4. (**E**) The proportion of male and female patients in different m6A score groups. (**F**) The difference in m6A scores between male and female patients. (**G**) Analysis of m6A score and immune checkpoints between the m6A score subgroups

The correlation between clinical characteristics and m6A score was assessed. living and stage T1-2 patients had lower m6A scores than those who had died or had more advanced disease (Fig. 7A and D). No differences in m6A scores were observed by sex (Fig. 7E F). The expression of chemokines, cytokines, and their receptors was assessed to understand the impact of m6A on HBV-related HCC. VTCN1, CD47, CD80, SIRPA, TNFRSF4, CD28, ADA, CD86, and LGALS9 expression were higher in the low-scoring m6A cluster, suggesting that these molecules may be potential therapeutic targets for m6A regulators in this group (Fig. 7G). Since HBV-related HCC is treated using multiple methods, the patient response to 138 different types of drugs extracted from the GDSC database was explored. Using the “pRRophetic” R package, each drug’s IC50 was predicted as a marker of its sensitivity. While 27 drugs demonstrated a lower IC50 in the low-scoring m6A group, 71 drugs were more sensitive for patients in the high-scoring m6A group (Supplementary Fig. 2). These results provide possible new targets for HCC treatment (Supplementary Fig. 3).

### M6A regulator expression in HBV-related HCC

**Fig. 8:**
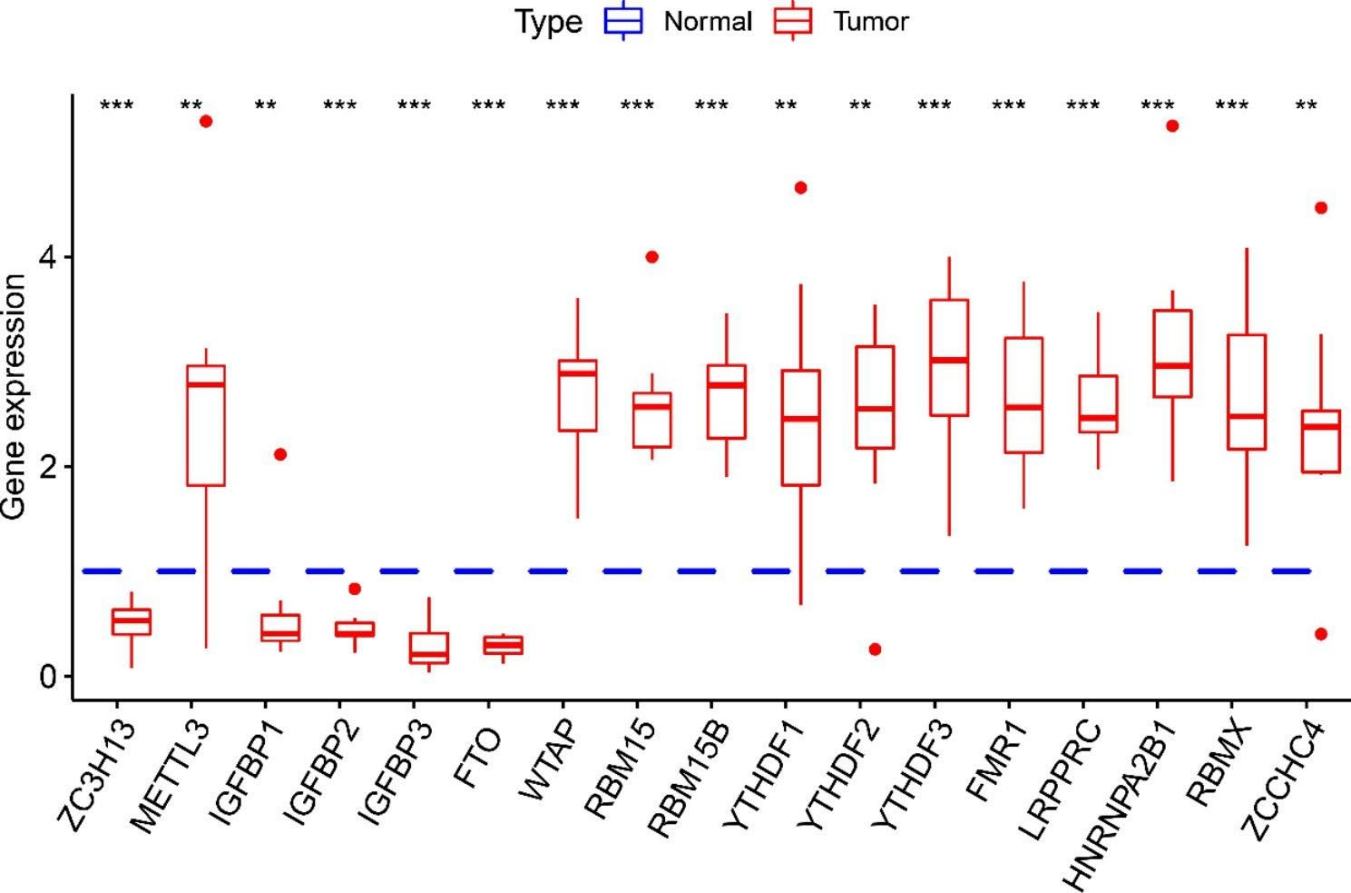
The expression of m6A in HBV-related patients

To further explore the expression of these 20 m6A regulators in HBV-related HCC, we investigated the differential mRNA expression of these genes was investigated by comparing HBV-related HCC and liver tissues using RT-qPCR. Consistent with what was observed in the database, METTL3, WTAP, RBM15, RBM15B, YTHDF1, YTHDF2, YTHDF3, FMR1, LRPPRC, HNRNPA2B1, RBMX, and ZCCHC4 were more highly expressed in tumor than normal tissues, while ZC3H13, IGFBP1, IGFBP2, IGFBP3, and FTO had reduced expression in tumor than normal tissues (Fig. 8).

## Discussion

Recently, a significant number of studies have investigated the correlation between m6A and HCC, giving important benefits to HCC patients in various clinical decision-making processes, including target therapy, immunotherapy, and prognosis evaluation [31, 32]. These studies have also enhanced our understanding of the biology and functions of HCC. Some studies have found that variations in m6A expression affect the TIME of HCC. Fang et al. reported that the way sh-METTL3 suppresses the polarization of Kupffer cells and the advancement of HCC is by regulating RBM14 expression through YTHDF1-dependent m6A modification [33]. Given the complexity and heterogeneity of HCC, it is important to continually study molecular markers that are associated with cancer prognosis using gene expression patterns, so as to uncover additional therapeutic targets.

HBV is one of the main causes of HCC and tumor immune escape is more likely to occur in HBV-associated HCC as a result of reduced viral antigen expression [34]. Immune cell changes contribute to alterations in the immune microenvironment of HBV-associated HCC [35]. HBV can induce m6A modification of the immune reactor-related protein, PTEN RNA, thus affecting the innate immune response and leading to immune escape [36]. RNA m6A modification not only affects the proliferation, metastasis, and prognosis of HCC but also impacts the prognosis of HBV-related HCC [37]. However, existing studies have not explored the correlation between M6A-related regulatory genes and the prognosis of HBV-associated HCC and only report on the role of m6A modification on disease outcomes. Thus, it is critical to determine the association between m6A-related regulatory genes and the prognosis of HBV-associated HCC. Tumor development and progression were historically thought to be regulated by genetic and epigenetic alterations in tumor cells. Recent studies have indicated that the purity of the TME also has a significant effect on tumor development [37]. To identify tumor immunophenotypes and novel therapeutic targets, it is necessary to comprehensively explore the heterogeneity and complexity of the TME.

Emerging evidence shows that m6A modification is associated with several pathological processes of malignancy, including cell proliferation, migration, and invasion [38]. Most studies are concentrated on one or two m6A regulators in the TIME, thus it is important to characterize the potential mechanism by which all the m6A regulators work together to mediate immune cell function [39]. Identifying the characteristics of different m6A regulators in the TIME will facilitate the development of more effective treatments.

Two m6A modification patterns in HBV-related HCC were established from the TCGA and GEO cohort based on m6A regulator expression. These two m6A modification patterns had distinct levels of immune infiltration. It has been shown that m6A modification patterns can predict tumor inflammation, genetic variation, and prognosis outcomes of patients with HBV-related HCC. This phenomenon may be related to changes in two metabolic patterns in the TIME, increased tumor cell expression, and/or enhanced anti-PD-1/PD-L1 immunotherapy responses [40]. The results of this study indicated that each m6A cluster was associated with a different pattern of immune infiltration. After extracting the DEGs from each m6A cluster, 20 m6A regulator genes were found to be differentially expressed, confirming that they are closely related to the TIME and prognosis of patients with HBV-related HCC. These findings help to define the TIME landscape associated with distinct m6A regulator expression patterns.

This study highlights a demand to develop a novel system for quantifying m6A modification patterns. To achieve this, a novel m6A scoring system was established to identify the m6A modification pattern of individuals with HBV-related HCC. The m6A score was highest in m6A cluster A and this cluster had significantly lower expression than other groups, suggesting that this score could serve as a reliable biomarker for m6A modification patterns.

Findings indicated that the m6A score is closely related to the TIME landscape of HBV-related HCC and that it may be a predictive factor for the therapeutic effect of the immune checkpoint. A low m6A score was closely related to immune checkpoints, including VTCN1, CD47, CD80, SIRPA, TNFRSF4, CD28, ADA, CD86, and LGALS9. Scores were also associated with different levels of treatment sensitivity.

However, this research has some limitations. It mainly centered on the predictive value of the m6A regulator, but further investigations using both in vivo and in vitro validation are necessary to completely understand the role of m6A modification in Hepatocellular Carcinoma related to HBV.

## Conclusion

This study demonstrated that m6A modification significantly affects tumorigenesis, TME, and outcomes of patients with HBV-related HCC. The m6A score was shown to play an important role in illustrating the heterogeneity and complexity of individual TIME. Comprehensive assessment of m6A patterns will enhance the future understanding of immune cell infiltration and inform individualized cancer therapy.

## Electronic supplementary material

Below is the link to the electronic supplementary material.

Supplementary Material 1: Supplementary Figs. 1, 2 and 3

## Data Availability

All raw data on hepatocellular carcinoma, which were included in the current study, can be downloaded from TCGA (https://portal.gdc.cancer.gov/).
