# Supplementary material for: Comprehensive analysis of m6A regulators associated with immune infiltration in Hepatitis B virus-related hepatocellular carcinoma
Source: BMC Gastroenterol. 2023 Jul 28;23:259. doi: 10.1186/s12876-023-02873-6 (PMC10385918; doi:10.1186/s12876-023-02873-6)
Supplement: Supplementary file 1 — Supplementary Material 1: Supplementary Figs. 1, 2 and 3 [file 12876_2023_2873_MOESM1_ESM.docx]

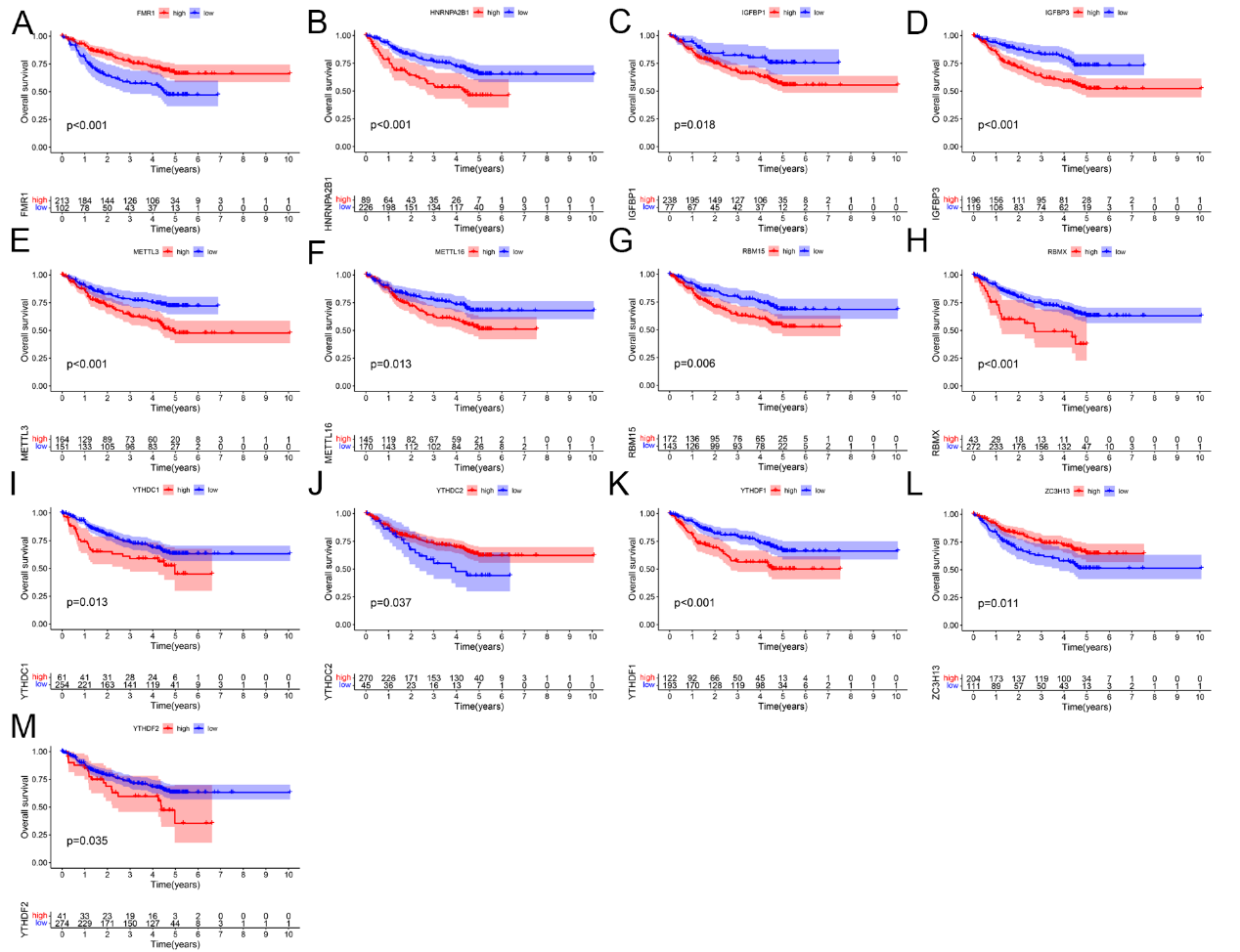


Supplementary Figure 1. Survival analysis of m6A regulators and overall survival of patients with HBV-related HCC.


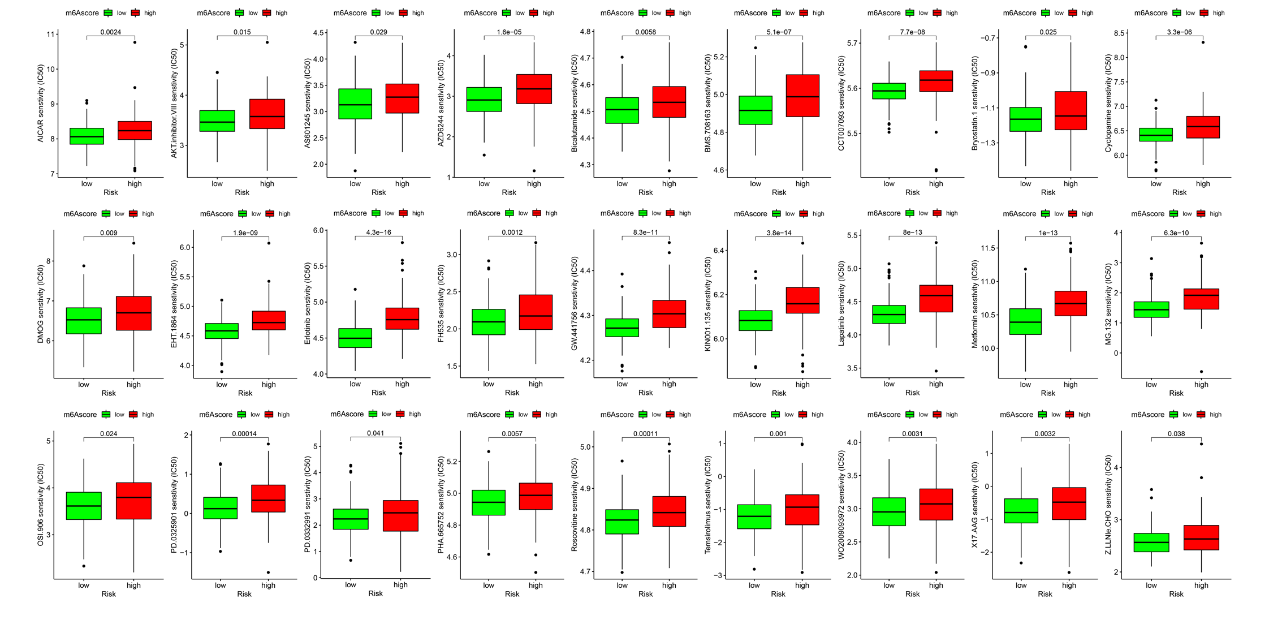


Supplementary Figure 2. Sensitivity of antitumor drugs with a lower IC50 in the low m6A score subgroup.


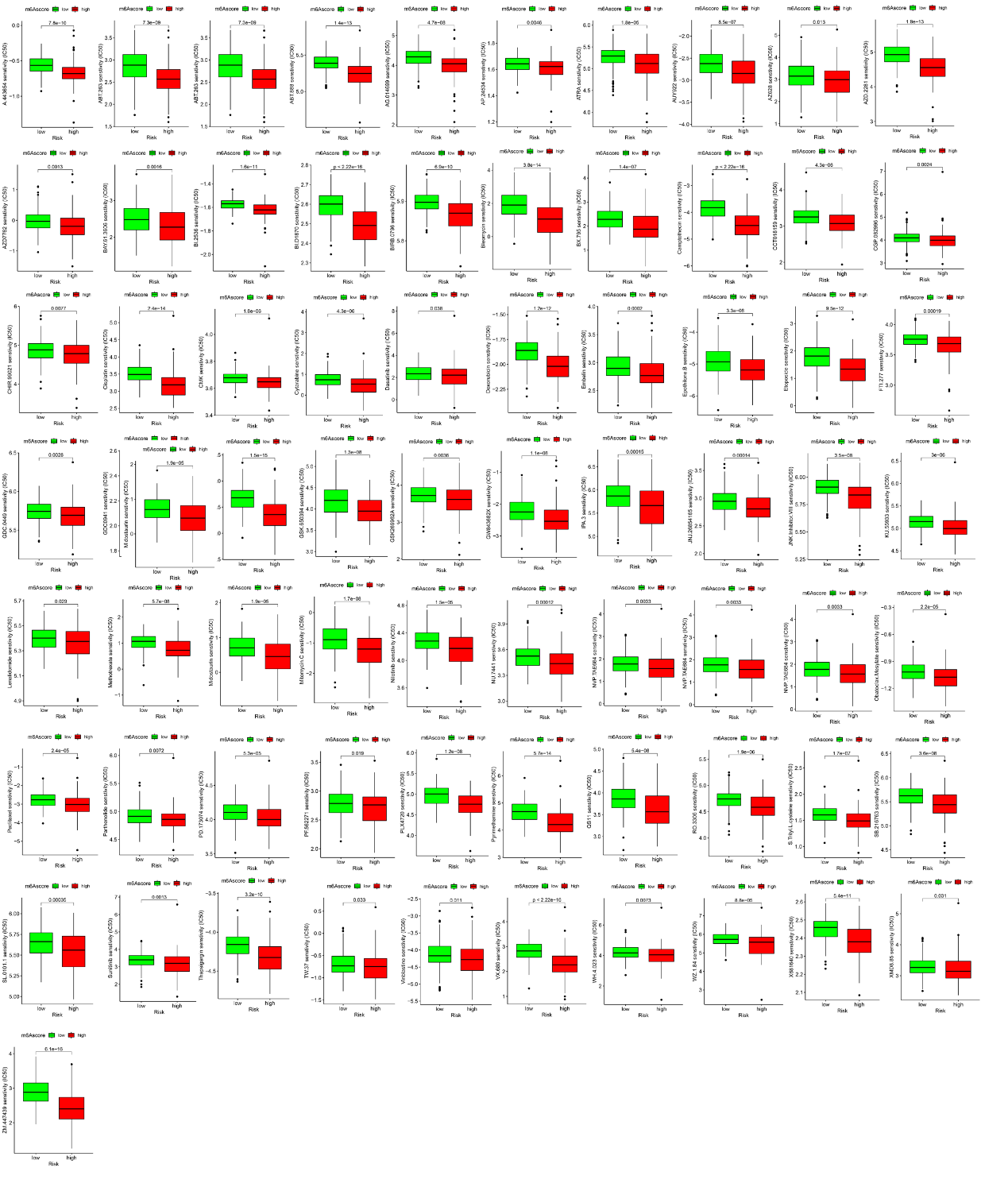


Supplementary Figure 3. The sensitivity of antitumor drugs with a lower IC50 in the high score subgroup.
